# Complete Genome Sequence of *Microbacterium* Bacteriophage Erla

**DOI:** 10.1128/MRA.01354-20

**Published:** 2021-01-21

**Authors:** Mark Milhaven, Erin Hastings, Danielle Brister, Leo Cevallos, Sriya Chilukuri, Arshia Diyya, Aman Garg, Rosaura Hernandez, Daniel Kelly, Karina Lazo, Jennifer Le, Garett Maag, Palak D. Marfatia, Rithik Mehta, Aram Nejad, Jade Porche, Alexander Queiroz, Daniel Sackett, Pablo Santos Molina, Taylor Slade, Minerva So, Karan Thakur, Angelica Urquidez Negrete, Sage Wackett, Sarah Weiss, Liam McCarthy, Keith Wheaton, Adam D. Rudner, John P. McCutcheon, Susanne P. Pfeifer

**Affiliations:** a School of Life Sciences, Arizona State University, Tempe, Arizona, USA; b Translational and Molecular Medicine Program, University of Ottawa, Ottawa, Ontario, Canada; c Center for Mechanisms of Evolution, Arizona State University, Tempe, Arizona, USA; d Department of Cellular and Molecular Medicine, University of Ottawa, Ottawa, Ontario, Canada; e Department of Biochemistry, Microbiology and Immunology, University of Ottawa, Ottawa, Ontario, Canada; f Center for Evolution and Medicine, Arizona State University, Tempe, Arizona, USA; Queens College

## Abstract

We characterized the complete genome sequence of *Siphoviridae* bacteriophage Erla, an obligatory lytic subcluster EA1 bacteriophage infecting Microbacterium foliorum NRRL B-24224, with a capsid width of 65 nm and a tail length of 112 nm. The 41.5-kb genome, encompassing 62 predicted protein-coding genes, is highly similar (99.52% identity) to that of bacteriophage Calix.

## ANNOUNCEMENT

The discovery of antibiotic agents in 1928 by Alexander Fleming has completely changed the way clinicians treat bacterial infections. However, with new therapeutic challenges resulting from antibiotic resistance, scientists have now turned to bacteriophages as promising clinical alternatives to antibiotics (1). Aiding this endeavor is the vast, though largely uncharacterized, diversity of bacteriophages across the globe (current estimates range in the order of 10^31^ [2]).

**FIG 1:**
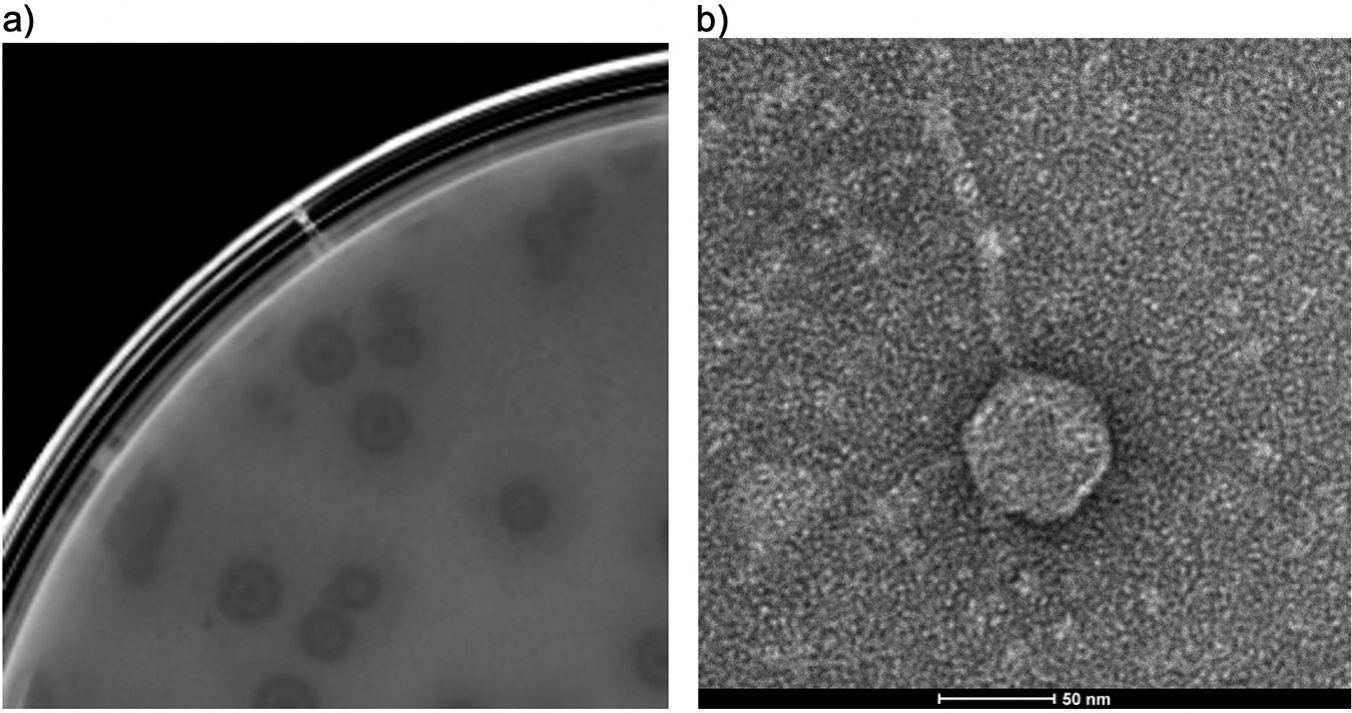
Characterization of the *Microbacterium* bacteriophage Erla. (a) Erla forms medium-sized plaques with a faint bullseye in the middle. (b) Transmission electron microscopy image showing Erla’s *Siphoviridae* morphology with an icosahedral capsid (diameter, 65 nm) attached to a 112-nm-long tail.

Here, we report the complete genome sequence of bacteriophage Erla. Erla was obtained from a soil sample collected at 16°C from a garden in Ottawa, Ontario, Canada (45.428778 N, 75.677246 W). Following the standard procedures outlined in the Science Education Alliance-Phage Hunters Advancing Genomics and Evolutionary Sciences (SEA-PHAGES) Discovery Guide (https://seaphagesphagediscoveryguide.helpdocsonline.com/home), the sample was mixed in an equal volume of peptone-yeast-calcium agar (PYCa) medium and shaken (250 rpm) at 30°C for 2 h. Next, the sample was filtered (pore size, 0.22 μm), mixed with 500 μl host cells (Microbacterium foliorum NRRL B-24224), and grown for 72 h with shaking (220 rpm). A 1-ml sample was filtered (pore size, 0.22 μm), and 100-μl samples of 10-fold serial dilutions were mixed with 300 μl host cells (Microbacterium foliorum NRRL B-24224) and 3.5 ml PYCa top agar. This mixture was incubated at 30°C for 2 to 3 days until plaques formed. Erla was purified by repeated rounds of plaque picking into phage buffer, serial dilution, and plaque assays of 100 μl of diluted phage, as described above. Amplification was performed by flooding “webbed” plates with phage buffer overnight at 4°C. Erla forms medium-sized plaques with a faint bullseye in the middle (Fig. 1a) and exhibits a *Siphoviridae* morphology with an icosahedral capsid (diameter, 65 nm) enclosing the double-stranded DNA, attached to a flexible, noncontractile tail (length, 112 nm; Fig. 1b).

In order to sequence Erla’s genome, DNA was extracted from the phage lysate using a phenol-chloroform assay, followed by ethanol precipitation. Next, a sequencing library was prepared using the NEB Ultra II FS kit and sequenced on an Illumina MiSeq instrument (150-bp single-end reads) to >3,300× coverage (976,546 reads). Following Russell (3), the raw reads were assembled using Newbler v.2.9 (4), resulting in a single linear contig 41,538 bp in size with a GC content of 63.4%, similar to the 68.7% GC content of its host, Microbacterium foliorum NRRL B-24224. The assembly was checked for completeness, accuracy, and genome termini using Consed v.29.0 (5). As neither read start buildups nor substantial variations in coverage were observed, Erla’s genome is most likely circularly permuted.

Following the SEA-PHAGES Bioinformatics Guide (https://seaphagesbioinformatics.helpdocsonline.com/home), Erla’s genome was annotated using DNAMaster v.5.23.5 (http://cobamide2.bio.pitt.edu). Sixty-two putative genes were identified using GLIMMER v.3.02 (6), GeneMark v.3.25 and v.4.28 (7), and Starterator v.381 (https://seaphages.org/software), corresponding to a gene density of 1.49 genes/kb. ARAGORN v.1.1 (included in DNAMaster) and v.1.2.38 (8) and tRNAscan-SE v.2.0 (9) were used to search for tRNAs and transfer-messenger RNA (tmRNA), but none were found. Functional assignments were made using BLASTp v.2.10.1 (10) and HHpred (11), leading to a putative function for 26 out of the 62 genes. TMHMM v.2.0 (12) and SOSUI v.1.11 (13) were used to gather further information on proteins of no known function, leading to the identification of two additional transmembrane proteins. Default parameters were used for all software.

Phamerator v.381 (14) was used to determine synteny among Erla and other bacteriophages previously sequenced as part of the SEA-PHAGES program, which supported the above functional assignments. Following the classification guidelines established by Hatfull et al. (15), Erla was identified as a *Microbacterium* subcluster EA1 bacteriophage. Multiple sequence alignments using Kalign v.1.04 (16) and BLASTn v.2.10.1 (11) indicated that Erla is most closely related to Calix (percent identity, 99.52%; GenBank accession number MN234163.1), Gelo (percent identity, 99.51%; MG962367.1), and Etta (percent identity, 99.51%; MK977697.1).

### Data availability.

Whole-genome sequencing data are available through NCBI’s Sequence Read Archive (BioProject accession number PRJNA488469; run accession number SRR13108336). The annotated genome assembly has been deposited in GenBank under accession number MW291026.
